# Impact of DNA extraction method and targeted 16S-rRNA hypervariable region on oral microbiota profiling

**DOI:** 10.1038/s41598-018-34294-x

**Published:** 2018-11-05

**Authors:** Fei Teng, Sree Sankar Darveekaran Nair, Pengfei Zhu, Shanshan Li, Shi Huang, Xiaolan Li, Jian Xu, Fang Yang

**Affiliations:** 1 Single-Cell Center, Qingdao Institute of Bioenergy and Bioprocess Technology, Chinese Academy of Sciences, Qingdao, Shandong 266101 China; 2 Department of Stomatology, Qingdao Municipal Hospital, Qingdao, Shandong 266101 China; 3 Guangdong Provincial Key Laboratory of Stomatology, Sun Yat-sen University, Guangzhou, 510055 China; 4 University of Chinese Academy of Sciences, No.19(A) Yuquan Road, Shijingshan District, Beijing 100049 P.R. China

## Abstract

Amplification and sequencing of 16S amplicons are widely used for profiling the structure of oral microbiota. However, it remains not clear whether and to what degree DNA extraction and targeted 16S rRNA hypervariable regions influence the analysis. Based on a mock community consisting of five oral bacterial species in equal abundance, we compared the 16S amplicon sequencing results on the Illumina MiSeq platform from six frequently employed DNA extraction procedures and three pairs of widely used 16S rRNA hypervariable primers targeting different 16S rRNA regions. Technical reproducibility of selected 16S regions was also assessed. DNA extraction method exerted considerable influence on the observed bacterial diversity while hypervariable regions had a relatively minor effect. Protocols with beads added to the enzyme-mediated DNA extraction reaction produced more accurate bacterial community structure than those without either beads or enzymes. Hypervariable regions targeting V3-V4 and V4-V5 seemed to produce more reproducible results than V1-V3. Neither sequencing batch nor change of operator affected the reproducibility of bacterial diversity profiles. Therefore, DNA extraction strategy and 16S rDNA hypervariable regions both influenced the results of oral microbiota biodiversity profiling, thus should be carefully considered in study design and data interpretation.

## Introduction

Oral microbiota are implicated in the aetiology of many oral and systemic diseases, such as dental caries, periodontal diseases, obesity and cancer^1–4^. Therefore, testing the association between oral microbiota structure with diseases is of great interest in clinical diagnosis and treatment^1^. One vital approach for this purpose is high-throughput sequencing of the 16S rRNA gene amplicons. This approach has allowed characterization of the microbiome diversity from human and environmental microbiomes with an unprecedented depth and coverage^5–8^. However, for human intestinal microbiota^9–13^ and environmental microbiota^14–17^, various factors such as DNA extraction method, targeted 16S rRNA hypervariable regions and sample handling environment can all greatly influence the resulted biodiversity profiles.

First of all, DNA extraction can be crucial to the success of microbiome sequencing^18–22^. The wide variation of cell membrane structures and compositions can pose a significant challenge to the efficient and bias-free extraction of genomic DNA^23^. In particular, cell lysis is an initial yet crucial step in DNA extraction procedures and typically includes physical, chemical and enzymatic disruption^14,24–26^. Physical disruption can increase the DNA yield, yet at the same time may potentially shear genomic DNA into small fragments which can lead to chimeric products in the final sequencing result^27,28^. Chemical and enzymatic lysis methods are less likely to damage DNA, but they can introduce bias in DNA extraction, due to the lower general applicability to all target organisms^26,29^. The oral microbiota are highly complex and heterogeneous, which consist of a wild array of Gram-positive and Gram-negative organisms. However, for oral microbiota, the impacts of these DNA extraction strategies on microbiome sequencing has not been critically assessed. As a result, how to rationally devise an experimental procedure to achieve an accurate and reliable representation of oral microbiota diversity via sequencing is not yet firmly established.

On the other hand, the choice of 16S rRNA hyper-variable regions targeted for sequencing, which have been the most widely used markers to assess the phylogenetic diversity of microbes, is an important decision to make. A frequent choice is the V1–V3 region, but its application has been so far limited to Roche/454 pyrosequencing platform (offering up to 750 bp single-end length reads)^30^. In addition, the V3–V4 hypervariable region has been targeted via the MiSeq platform (which can produce single-end reads of 350 bp), which can allow for more accurate and cost-effective characterizations of microbiome samples^30–32^. The V4-V5 region is also a traditionally employed hypervariable region in 16S rRNA based microbial biodiversity profiling studies^33,34^. Previous studies have shown that the choice of particular hypervariable region targeted in 16S rRNA sequencing can significantly alter the perceived structure of microbial community^16,17,35–37^. Therefore, critical assessment of the choice of hyper-variable regions will be important, so as to minimize distortion and conflicts in sequence-based analysis and comparison of oral microbiota.

A number of studies have investigated the effects of various factors on 16S rRNA gene based microbiome profiles (e.g., gut microbiota studies^38^, human microbiome mock sample^30^ and saliva sample^39^), such as sample storage prior to DNA extraction^40^, DNA extraction procedure^22,41–43^, primers^13,35,44^ and the sequencing platform used^13^. However, frequently no consensus conclusions were reached on how particular factors influence the results. For instance, in the case of DNA extraction method, Lazarevic *et al*.^39^ and Vesty *et al*.^43^ concluded that no significant difference in diversity indices was found between different extraction methods, although the mechanical lysis method revealed higher operational taxonomic unit (OTU) richness. On the contrary, another study found that the global community structure and relative abundance of individual taxa are both affected by DNA extraction method^35^. Except for such controversy, for human oral microbiome, such investigations have so far been limited to DNA extraction methods^22,39,43,45^. Therefore, efforts to critically evaluate how individual or combinations of the factors affects the observed structure of oral microbiota are of value.

Here, employing a mock oral bacterial community with a predetermined equal composition, we systematically evaluated the impacts of each or combinations of three factors including DNA extraction method, hypervariable regions and technical reproducibility on oral microbiome sequencing. Our aim is to quantitatively assess the contribution of each factor to the sequencing results. By providing the evidence for rational decision making for each of the factors, the findings should guide the design of experiments and workflows towards a complete, accurate and reliable portrait of the human oral microbiota.

## Materials and Methods

### Mock community construction

**Figure 1:**
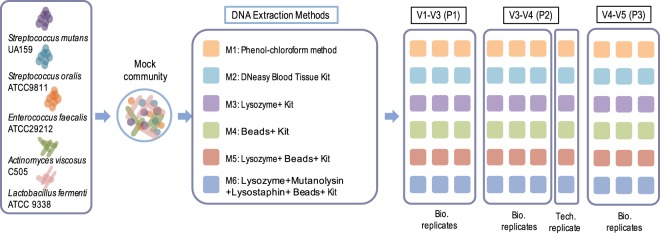
Overall workflow of the study. Mock community samples consisting of five oral strains were sequenced via six DNA extraction methods and followed by amplification of three 16S rRNA gene target regions. The results of subsequent 16S rDNA sequencing on the MiSeq platform were quantitatively compared. For each of the six samples (each of which underwent one of the six DNA extraction method), triple biological replicates (“Bio. replicates”) for each of the amplification regions were sequenced. In addition, for the amplification region of V3-V4 (P2), one technical replicate (“Tech. replicate”) for each of the six samples was sequenced. All the 54 biological samples were processed by the same experimenter (Experimenter A) and sequenced on the same sequencing batch (Batch A), while six technical replicates were processed by the other experimenter (Experimenter B) and on the other batch (Batch A).

Mock communities were prepared by mixing five representative oral bacteria (*Streptococcus mutans* UA159, *Streptococcus oralis* ATCC9811, *Actinomyces viscosus* C505, *Enterococcus faecalis* ATCC29212 and *Lactobacillus fermentum* ATCC9338; Table 1; Fig. 1). Cells cultivated in liquid medium were collected by centrifugation and then re-suspended in PBS to reach a cell density of 10^9^ cells per ml. The cell density was determined by bright-line counting chamber (Hausser Scientific, Horsham, USA). Then a mock community was prepared by mixing equal volumes of cell suspensions of the five bacteria. After centrifugation for 10 minutes at 7,500 rpm, the pellet of cellular mixture was re-suspended in 500 μl TE buffer and frozen at −80 °C.

**Table 1:** Information of bacterial strains used in constructing the oral mock community.

Type strains	Gram-strain	Condition	Medium	Copy number
*Streptococcus mutans* UA159	+	anaerobic	BHI	5
*Streptococcus oralis* ATCC9811	+	anaerobic	BHI	4
*Actinomyces viscosus* C505	+	anaerobic	BHI	3
*Enterococcus faecalis* ATCC29212	+	anaerobic	BHI	4
*Lactobacillus fermenti* ATCC 9338	+	anaerobic	MRS	5

### DNA extraction

Six DNA extraction methods commonly used in human microbiome studies were chosen for the comparison (Table 2). DNA was extracted from the mock community in triplicates, in parallel with a negative control (PBS buffer). DNA concentrations were estimated by PicoGreen dsDNA quantitation kit (Invitrogen, Carlsbad, USA). Details on those six DNA extraction methods were described as below. It is noted that except for M1, the cell lysis method prior to DNA extraction is the only difference among the five DNA extraction methods from M2 to M6.

**Table 2:** Features of the six DNA extraction methods tested in this study.

Method	Cell lysis	DNA purification
M1	Protease K	Phenol-chloroform purification and isopropanol precipitation
M2	Protease K	Silica column (DNeasy Blood Tissue Kit)
M3	lysozyme	Silica column (DNeasy Blood Tissue Kit)
M4	bead beating	Silica column (DNeasy Blood Tissue Kit)
M5	lysozyme+bead beating	Silica column (DNeasy Blood Tissue Kit)
M6	lysozyme+mutanolysin+lysostaphin+bead beating	Silica column (DNeasy Blood Tissue Kit)

### Method1- Phenol:chloroform

A 500 μl aliquot of cells was added to a tube containing an equal volume of lysis buffer. Then 30 μl of proteinase K (20 mg/ml, Qiagen, USA) and 75 μl of 10% SDS were added to the mixture, which was then incubated overnight at 53 °C in a shaking water bath. After the addition of 200 μL of 5 M NaCl and then 10 min of incubation on ice, 800 μL of buffer-saturated phenol was added. The tubes were vortexed at the maximum speed and then centrifuged for 10 min at 13,000 rpm. The aqueous phase was transferred to a new sterile centrifuge tube, to which a second 800 μL of buffer-saturated phenol was added. The tubes were vortexed and centrifuged again for 15 min at 13,000 × g. After the upper phase was recovered, an equal volume of chloroform/isoamyl alcohol (24:1) was added. The tubes were vortexed and centrifuged again for 15 min at 13,000 x g. The supernatant from the tube was transferred to a new tube, where 400 μl of isopropanol was added. The tube was then incubated for 10 min at room temperature and centrifuged for 15 min at 13,000 rpm. The supernatant was discarded and the DNA pellets were washed twice with 250 μl of 70% ethanol. Once dried, DNA was dissolved in 100 μl of double-distilled water^46,47^.

### Method2-[DNeasy^®^ Blood + Tissue kit]

The DNeasy^®^ Blood & Tissue DNA kit (Qiagen Valencia, CA) was used based on manufacturer’s instruction. Briefly, cells were harvested from a 500 ul aliquot of culture in a microcentrifuge tube by centrifugation for 10 minutes at 7500 rpm. A buffer was added to re-suspend the pellets and the mixture was incubated for 30 min at 37 °C. Then 20 μl of Proteinase K was added and the mixture was incubated overnight at 56 °C. After these procedures, 200 ml AL buffer and 100 ml of ethanol were added. The rest of isolation protocol was performed based on the DNeasy^®^ Blood & Tissue DNA kit^46,48^.

### Method3-Chemical/enzymatic lysis + [DNeasy^®^ Blood + Tissue kit]

In this method, an extra enzymatic lysis step was added prior to the application of DNeasy^®^ Blood and Tissue kit (Qiagen Valencia, USA). Specifically, samples were mixed with 50 μl of lysozyme (final concentration 20 mg/ml, Sigma-Aldrich) and incubated for 1 hour at 37 °C. After this step, the mixture was supplemented with 20 μl of Proteinase K (20 mg/ml, Qiagen, USA) and incubated overnight at 56 °C. Then 500 μl AL buffer and 500 ml of ethanol were added to the lysate and the genomic DNA was purified according to the manufacturer’s instructions^48,49^.

### Method4-Bead beating + [DNeasy Blood + Tissue kit]

The original sample (500 μl) was transferred into a clean Bead-Beating-Tube (2 ml Eppendorf tube), and 600 mg of 0.1-mm-diameter zirconia-silica beads (BioSpec, Bartlesville, USA) were added to the mixture. Cells were then mechanically disrupted for 3 minutes and 26 Hz at room temperature in a Qiagen Tissue Lyser LT. Then the mixture was centrifuged at 13,000 rpm for 5 min and 360 μl of the crude lysate was transferred into a new tube. The remaining steps were performed using DNeasy® Blood & Tissue Kits according to the manufacturer’s instructions^48,50,51^.

### Method5-Chemical/enzymatic lysis + Bead beating + [DNeasy Blood + Tissue kit]

In this method, an extra step that consists of lysozyme-based enzymatic lysis and beating of zirconia-silica beads (BioSpec, Bartlesville, OK) was applied prior to the usage of DNeasy^®^ Blood and Tissue kit (Qiagen Valencia, USA) as described above. Samples were transferred into clean bead beating tubes (2 ml Eppendorf tube), and 50 μl of lysozyme (20 mg/ml, Sigma-Aldrich, USA) was added to a 500 μl aliquot of cell suspension followed by incubation for 1 hr at 37 °C. Then, 600 mg of 1 mm diameter zirconia-silica beads (BioSpec, Bartlesville, USA) were added to the lysate and the cells were subjected to bead beating using a Qiagen Tissue Lyser LT at 36 Hz for 3 min. Further isolation and purification of the total genomic DNA from lysates were conducted using the DNeasy^®^ Blood and Tissue kit according to the manufacturer’s instructions^48,49,51^.

### Method6-[Chemical/Lytic-Enzyme-Cocktail master-mix lysis] + Bead beating + [DNeasy Blood and Tissue kit]

A two-step cell lysis procedure was employed before the use of DNeasy^®^ Blood and Tissue kit (Qiagen Valencia, USA). Firstly, sample suspensions were kept on ice while a Lytic-Enzyme Cocktail was prepared. Freshly prepared 100 μl of Lytic-Enzyme-Cocktail master mix, including 50 μl of lysozyme (10 mg/ml, Sigma-Aldrich, USA), 6 μl of mutanolysin (5 KU/ml, Sigma-Aldrich, USA) and 3 μl of lysostaphin (4000 U/ml, Sigma-Aldrich), was added to samples and incubated at 37 °C for 45 min. Secondly, 600 mg cleaned and dry 0.1 mm diameter zirconia-silica beads were added to the lysate. Samples were then subjected to bead beating for 3 min at room temperature in a Qiagen Tissue Lyser LT (36 Hz). The remaining DNA extraction procedures were performed using the DNeasy^®^ Blood and Tissue kit according to the manufacturer’s protocol^26,48,49^.

### Evaluation of DNA yield and quality from the mock samples

DNA yield was determined fluorometrically using the High Sensitivity dsDNA kit (Invitrogen, CA, USA) on a Qubit® Fluorometer 1.0. DNA purity was assessed by measuring absorbance ratios spectrophotometrically on a NanoDrop® ND-1000 (NanoDrop Technologies, DE, USA), and the measurement includes A260/280 nm for protein contamination and A260/230 nm for salt and phenol contamination.

### DNA standard preparation and quantitative real-time PCR

The known DNA concentration standards were prepared from the PCR products of genomic DNA. Three biological replicates of 10-fold serially diluted DNA standards from the PCR products of *Prevotella Veroralis* JCM6290 was used for the standard curve generation to quantify total bacteria. Quantitative real-time PCR assays were performed to quantify the total bacteria content of the extracted nucleic acids using Roche LightCycler 480II. Each qPCR was performed in a reaction volume of 20 μl containing 0.5 μM of each primer (Bac-16s-F2: 5′-TTAAACTCAAAGGAATTGACGG, Bac-16s-R2: 5′-CTCACGRCACGAGCTGACGAC)^52^, 1 μl of genomic DNA, 10 μl of 2 × SYBR Premix EXTaq mix (Takara) and 5 μl sterilized DNase-RNase-free water in MicroAmp fast optical 96-well reaction plates (Applied Biosystems, USA) with adhesive sealing. The qPCR parameters were 95 °C for 10 min, 45 cycles of three amplification steps including: 95 °C for 15 s, 60 °C for 20 s and 72 °C for 15 s, and final cooling at 25 °C for 1 min. Reaction specificities were confirmed by melting curve analysis with a progressive increase in temperature from 65 to 94  °C at a 1 °C/sec transition rate and continuous fluorescence acquisition. Each qPCR reaction was performed in triplicate. A standard curve of each primer was conducted by measuring three 10-fold series diluted DNA standards (i.e., the targeted DNA fragment cloned into the plasmid of pMD 19 T) and three negative controls (ddH2O as the template) were included within every experiment. Gene copy number was calculated automatically based on the standard curve of each primer system using LightCycler 480 Software 1.5 (Roche, USA).

### Targeted 16S rRNA amplification and copy number identification

Amplicon libraries of three chosen hyper-variable regions including V1-V3, V3-V4, and V4-V5 in 16S rRNA genes were prepared in triplicates from each extracted DNA sample, and barcode sequenced using the 2 × 300 PE Illumina MiSeq platform^23^ (Table 3; Fig. 1). Copy numbers of 16S rRNA of the reference bacteria were identified based on the Ribosomal RNA Operon Copy Number Database (https://rrndb.umms.med.umich.edu/ ^53^; and the NCBI genome database (http://www.ncbi.nlm.nih.gov/sites/genome; Table 1).

**Table 3:** The 16S ribosomal RNA hypervariable regions tested in this study.

Region	Forward sequence	Reverse sequence
V1-V3 (P1)	TGGAGAGTTTGATCCTGGCTCAG	TACCGCGGCTGCTGGCAC
V3-V4 (P2)	CCTACGGRRBGCASCAGKVRVGAAT	GGACTACNVGGGTWTCTAATCC
V4-V5 (P3)	GTGYCAGCMGCCGCGGTAA	CTTGTGCGGKCCCCCGYCAATTC

### Evaluation of technical reproducibility

With respect to the technical reproducibility, we randomly chose six samples that targeted the V3-V4 hypervariable regions, each of which was processed via one of the six DNA-extraction methods tested (Fig. 1). Six mock samples, one for each extraction method, were processed in duplicates by two independent experimenters. Results were compared between these two batches that followed an identical experimental procedure, in order to assess the technical reproducibility of the pipeline.

### Bioinformatic analysis

Mothur was used for quality assessment of raw reads^54^, which were assembled and screened based on the minimum length of 250 bp and a maximum length of 550 bp. The SILVA 16S rRNA database was used for sequence alignment. Taxa were assigned for each sequence to the oral “CORE” reference database^55^ with a confidence threshold of 80%. Microbial community diversity, including α and β diversity, was analyzed at the species level and the genus level. Simpson and Shannon diversity indices that measure richness and evenness were employed to estimate diversity within each of the experimental protocols above. For β diversity, Bray-Curtis and Euclidean distance measures were applied to assess the dissimilarity among observed microbiome structures. The observed relative abundance of each taxon was estimated by counting the number of reads for each taxon and then normalizing by the total number of reads per sample. Since an equal number of each kind of bacterial cells was present in the mock community, the expected relative abundance of each taxon was calculated based on the observed relative abundances of 16S rRNA gene reads as normalized by the copy number of 16S rRNA gene in each species.

### Statistical analysis

To assess the influence of DNA extraction procedures on DNA quantity (i.e. DNA yield), quality (i.e. A260/A280 and A260/A230) and stability (i.e. total bacterial quantification), ANOVA (one-way analysis of variance) was employed with Tukey-Kramer post-hoc test for multiple pairwise comparisons. The p-values were adjusted for multiple testing according to Bonferroni adjustment. Based on the Bray-Curtis metric, PCoA (Principal Component Analysis) was performed to visualize the level of dissimilarity among the observed microbiome structures from the various experimental procedures. Adonis analysis was applied to determine the significance (*p*-value) and strength (an R^2^ value) of a given grouping factor in determining the variation of distances, with pairwise Adonis for multiple pairwise comparisons. A hierarchical cluster analysis was performed based on the Bray–Curtis distance for the one expected microbiota and 54 mock communities via the complete linkage method. A dendrogram was employed to visualize the results. To compare the quantitative data in α and β diversity analysis and biomarker selection, the Kruskal-Wallis rank sum test was used with false discovery rate (FDR) adjustment for multiple pairwise comparisons. Regarding α diversity analysis, the Shannon or Simpson indices were compared among the different groups, and between the expected microbiota structure and the observed ones. The extent of changes in α-diversity indices was also assessed among the different groups. Procrustes rotation analysis was performed between two subsets of transformed data to test the degree of difference among distinct batches or experimenters. For Procrustes analysis, *p* values were generated using 1,000 Monte Carlo simulations.

## Results

In this study, based on a mock oral microbiota consisting of five oral bacterial species of equal abundance, we compared the 16S amplicon sequencing results from six frequently employed DNA extraction procedures and three pairs of widely used 16S rRNA hypervariable regions (Tables 1, 2; Fig. 1). There are 60 mock samples in total, which yielded 980,511 bacterial 16S rRNA gene sequences that passed stringent quality control, averaging 16,342 reads per sample.

### Influence of DNA Extraction Methods on the Quantity, Quality and Stability of DNA

**Figure 2:**
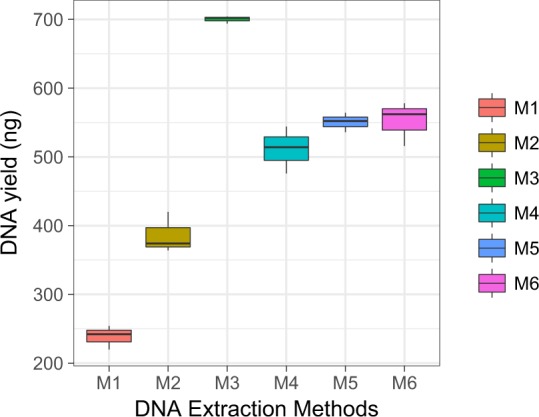
DNA yield of samples processed by the six DNA extraction methods. All the DNA extraction methods produced high-quality metagenomics DNA from the mock community samples, but the difference in methods affected the final extracted DNA yield significantly (*p* < 0.01, ANOVA). The highest DNA yield was found in the M3 [lysozyme + kit] extraction method (M3, *p* < 0.001, *post-hoc* test). The addition of beads (M4, M5, and M6; *p* < 0.001, *post-hoc* test) significantly reduced the final concentration of DNA, and no significant difference was found in DNA yield between these three methods (*p* > 0.05, *post-hoc* test).

Using gel electrophoresis, we confirmed the presence of metagenomic DNA from all the six DNA extraction methods. All the DNA extraction methods were able to produce high-quality metagenomic DNA from the mock community samples, but the difference in methods significantly affected the concentration of final extracted DNA (*p* < 0.01; Fig. 2). Pairwise comparisons showed that the traditional Phenol:chloroform-based method (M1) yielded the lowest amount of metagenomic DNA, which was followed by Kit DNA extraction method (M2) with a significant difference (*p* < 0.001; Fig. 2). The highest DNA yield was found in lysozyme + kit DNA extraction method (M3; all *p* values < 0.001; Fig. 2). On the contrary, the addition of beads (M4; *p* < 0.001) or the combination of [beads + lysozyme] (M5; *p* < 0.001) or [beads + Lytic- Enzyme Cocktail] (M6; *p* < 0.001) resulted in a significantly reduction in the final DNA concentration yielded (Fig. 2), although there was no significant difference in the amount of DNA yielded among these three methods (*p* < 0.05; Fig. 2).

Moreover, the impact of DNA extraction methods on DNA quality was assessed via A260/A280 and A260/A230. There was a significant difference between the mean A260/A280 ratios of DNA extracts between M1 and the other five methods (*p* < 0.05). In fact, all the A260/A280 ratios were between 1.8 and 2, which are generally indicative of “pure” DNA, except for M1 (Table 4)^56^. Similarly, the protocols that included commercial kits (M2-M6) had significantly less residual carryover than the non-commercial method (M1; *p* < 0.01), and the former (M2-M6) all produced DNA with the mean A260/A230 ratio between 1.8 and 2 (Table 4)^56^. These results suggested that, in this case, the commercial kit methods produced DNA with higher and more consistent purity, while cell lysis methods have no effect on DNA purity.

**Table 4:** The properties of DNA extracted by six DNA extraction procedures (M1 to M6).

Method	A260/A280	A260/A230	Total bacterial copy number (log)
M1	1.78 ± 0.03	1.95 ± 0.06	6.6 ± 0.25
M2	1.86 ± 0.01	2.12 ± 0.04	7.25 ± 0.13
M3	1.87 ± 0.02	2.13 ± 0.03	8.51 ± 0.15
M4	1.86 ± 0.01	2.12 ± 0.05	7.76 ± 0.11
M5	186 ± 0.02	2.11 ± 0.04	7.67 ± 0.15
M6	1.85 ± 0.02	2.08 ± 0.04	7.65 ± 0.13

Furthermore, to compare the stability of DNA derived from the extraction methods, total bacterial DNA copy number was quantified via the real-time qPCR (Table 4). Difference in methods significantly affected the total bacterial DNA copy numbers (*p* < 0.05, ANOVA). All the five methods using the commercial kit (M2-M6) resulted in increased total bacterial copy numbers, as compared with the non-commercial method (M1; all *p* < 0.01). Remarkably, the sole addition of lysozyme (M3) produced the highest total bacterial copy numbers. On the other hand, the addition of beads (M4, M5 and M6) appeared to be less effective than M2 (all *p* < 0.01) in increasing the total bacterial copy numbers (no significant difference in DNA yield among M4, M5 and M6; *p* > 0.05). Thus the pattern of total bacterial DNA copy numbers across the methods is largely consistent with that of the DNA yields (Table 4), suggesting the stability of DNA produced by the methods tested.

### Impact on Observed Microbiota Structure

**Figure 3:**
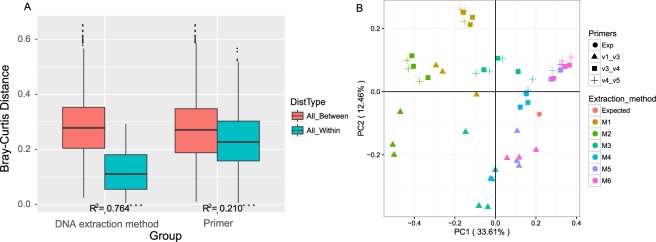
Impact of experimental procedures on the variation of mock oral microbiota. Bray-Curtis distance was calculated and compared between all pairs of samples collected from the expected microbiota and the 54 mock ones. (**A**) The perceived microbiome structure within and between DNA extraction methods and choice of hypervariable regions. DNA extraction method (R^2^ = 0.764, *p* < 0.001, Adonis) dominated the effect on oral microbiota structure over choices of primer sets (R^2^ = 0.21, *p* < 0.001, Adonis). (**B**) PCoA plot of the expected and all the 54 mock communities with the Bray-Curtis distance. For the DNA extraction methods, apart from the similar performance between M5 and M6 (*p* = 0.513, Kruskal-Wallis test), the other extraction methods produced significantly different oral microbiota structures (all *p* < 0.01, Kruskal-Wallis test). As for the choice of primer sets, results for oral microbiota from P2 (V3-V4) and P3 (V4-V5) were similar (*p* = 0.825, Kruskal-Wallis test), and both of them were distinct from P1 (V1-V3) samples (*p* < 0.01, Kruskal-Wallis test).

To identify microbiota features that are associated with the experimental procedures, the observed and expected microbiota structures were clustered via Bray-Curtis-based PCoA (Fig. 3A,B). Both the DNA extraction method and the choice of hypervariable region exhibited significant influence on microbiota community structure (*p* < 0.001; Fig. 3A), and the effect of the former (R^2^ = 0.764) was stronger than that of the latter (R^2^ = 0.210; Fig. 3A). Those two factors collectively explained up to 97.4% variation of microbiota structure (no inter-dependence found between the two factors; Fig. 3A). From the DNA-extraction-method point of view, apart from the similar performance of M5 and M6 (*p = *0.513), other extraction methods produced significantly different oral microbiota structures (pairwise comparisons; all *p* < 0.01; Fig. 3B). With respect to the choice of the hypervariable region, observed structure for oral microbiota from P2 (V3_V4) and P3 (V4_V5) were similar (*p* = 0.825), and both of them were distinct from P1 (V1_V3) (*p* < 0.01; Fig. 3B).

### Impact on Taxonomic Identification

**Figure 4:**
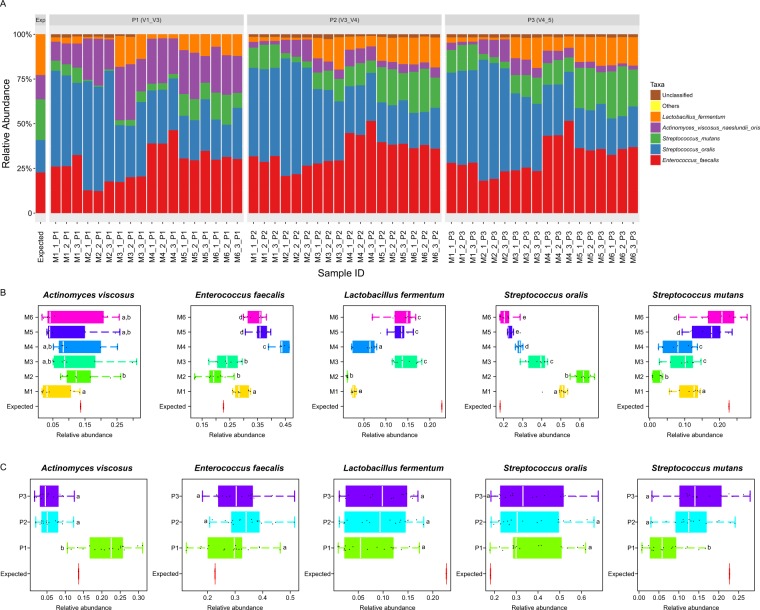
Comparison on the relative abundance of the five bacterial species in the mock community between the observed and the expected microbiome structures. (**A**) Relative abundance of the five bacterial species from each sample. (**B**) Comparison among the extraction methods. (**C**) Comparison among the targeted hypervariable regions. *E*. *faecalis* and *S*. *oralis* were over-represented as compared to the expected relative abundance, while *L*. *fermentum* and *S*. *mutans* were under-represented. *A*. *viscosus* was not consistently represented as compared to the expected result. M5 and M6 exhibited the best concordance with the expected distribution, followed by M3. P1 (V1_V3) performed better by producing results closer to the expected relative abundance of *A*. *viscosus* than V3-V4 and V4-V5, whereas V3-V4 and V4-V5 generated results that are in closer concordance with the expected abundance of *S*. *mutans*. Distinct letters denote significant difference between groups (*p* < 0.05, Wilcoxon sum-rank test).

Taxonomic compositions of the observed microbiota were compared with the expected ones to test whether our protocols can accurately measure community composition. Interestingly, none of the procedure tested here was able to perfectly recapitulate the actual composition. In general, *Enterococcus faecalis* and *Streptococcus oralis* were over-represented (as compared to the expected relative abundance), while *Lactobacillus fermentum* and *Streptococcus mutans* were under-represented at the species level (Fig. 4A). *Actinomyces viscosus* was not consistently represented as compared to the expected, being underestimated in P1 (V1_V3) and M1 while overestimated in P2 (V3_V4) and P3 (V4_V5) and those DNA extraction methods except M1 (Fig. 4A).

Specifically, (*i*) M5 and M6 exhibited the highest concordance with the expected distribution of mock-community members except for *Enterococcus faecalis*, and they were followed by M3 (Fig. 4B); (*ii*) Although quantification of *Enterococcus faecalis* was more accurate in M2 and M3, M2 produced the largest bias on the abundance of *Lactobacillus fermentum*, *Streptococcus oralis* and *Streptococcus mutans* (Fig. 4B); (*iii*) the largest distortion of the community composition was found in M1 and M4 respectively, i.e., the over-representation of *Actinomyces viscosus* in M1 and the under-representation of *Enterococcus faecalis* in M4 (Fig. 4B). The hypervariable region pairs produced different results only for *Actinomyces viscosus* and *Streptococcus mutans* (all *p* values < 0.05; Kruskal-Wallis test). Intriguingly, P1 (V1_V3) offered better performance than P2 (V3_V4) and P3 (V4_V5) for *Actinomyces viscosus*, whereas P2 (V3_V4) and P3 (V4_V5) exhibited closer concordance with the expected abundance of *Streptococcus mutans* (*p* < 0.05, P1 (V1_V3) vs. P2 (V3_V4) and P1 (V1_V3) vs. P3 (V4_V5); Fig. 4C).

### Impact on Microbial Diversity Assessment

**Figure 5:**
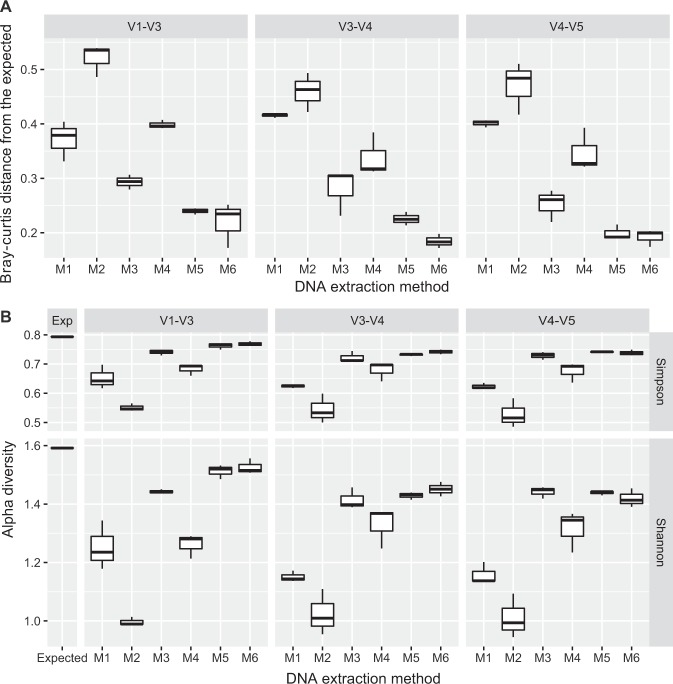
Comparison of α- and β- diversity between the observed and the expected community structures from each experimental procedure. (**A**) Box plots of Bray-Curtis distances between the expected and the observed microbiome diversity. The highest reproducibility of oral microbiota structure was found in those featuring combination of mechanical and enzymatic lysis methods (M5 and M6), followed by M3, M4, M2 and M1. (**B**) Both Shannon and Simpson indices representing α diversity were significantly different among the DNA extraction methods (*p* < 0.001, Kruskal-Wallis test), yet no difference was found among hypervariable regions on both indices (*p* > 0.05, Kruskal-Wallis test). Regardless of the primer sets chosen, α diversity indices from M5, M6 and M3 were the closest to the expected microbiota structure (*p* > 0.05, Kruskal-Wallis test).

To quantitatively evaluate which method generates an oral microbiota structure that is the most similar to the expected structure, we calculated the Bray-Curtis distances between expected and observed bacterial profiles for each of the protocols (Fig. 5A). Kruskal-Wallis analysis showed that DNA extraction methods exerted a substantial effect on the β diversity of oral microbiome (*p* < 0.001), whereas the hypervariable regions had little effects (*p = *0.661). Based on pairwise comparisons, the most reliable extraction method for reproducing the original microbiome structure was found in the combination of mechanical and enzymatic-lysis DNA-extraction methods (M5 and M6), followed by M3, M4, M2 and M1 (all *p*-values < 0.05; Fig. 5A). Moreover, hierarchical clustering of the microbial profiles from different experimental protocols was performed (Fig. S1). The formation of two major clusters suggested again that the difference between the DNA-extraction procedures outweighed the variations due to choice of hypervariable regions (Fig. S1). Moreover, protocols that included enzymatic-mechanical-lysis steps in their extraction procedure (e.g., M5 and M6) were clustered with the expected microbiome structure, thus they produced better microbial diversity representation than those methods without an extra step in the procedure (e.g., M1, M2, and M3; Fig. S1). In terms of the hypervariable regions, samples from P2 (V3_V4) and P3 (V4_V5) tend to cluster closely with the expected microbiota structures (Fig. S1).

Furthermore, we assessed the impact of the various methods on the α diversity as represented by Shannon and Simpson indices. The results were in agreement with the β diversity analysis: both indices were significantly different among the various DNA extraction methods (*p* < 0.001, Kruskal-Wallis test; Fig. 5), whereas no difference was found among the hypervariable regions (*p* > 0.05, Kruskal-Wallis test; Fig. 5). In fact, when compared with the other methods, α diversity indices detected by M5, M6 and M3 were the closest to the expected ones (pairwise comparisons; all *p*-values < 0.05, Fig. 5).

### Reproducibility of the Illumina-sequencing platform

**Figure 6:**
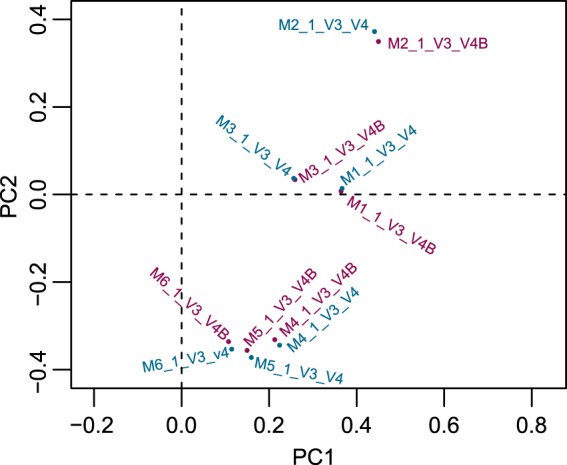
Validating the reproducibility of bacterial profile between the various experimental and sequencing batches. In the P2 (V3_V4) dataset, twelve mock microbiota were processed by two operators using six DNA extraction methods and then sequenced in the two batches on the Illumina MiSeq platform. Procrustes analysis showed that the agreement of bacterial profiling among technical replicates was excellent based on the Euclidean distances (*p* < 0.001, Monte-Carlo permutation test).

To evaluate the reproducibility of the DNA extraction methods in microbiota sequencing, we performed “replicated” samples of the mock community for those six DNA extraction methods in P2 (V3_V4; Fig. 1), where DNA was extracted by two independent experiments and sequenced on two batches of the Illumina MiSeq platform. PCoA plots of those twelve samples, for which β diversity was estimated using the Euclidean distance matrices, were constructed (Fig. 6). Specifically, one sample for the P2 (V3-V4) region was chosen from each of the six DNA extraction methods, and each of the six samples underwent two experimental procedures. Each procedure involved one distinct technician and one separate sequence batch. Analysis of the data using Procrustes test revealed a good agreement between results from the different experimenters, and also for sequencing performed among different batches (*p* < 0.001, Monte-Carlo permutation test; Fig. 6).

## Discussion

The human body is home to many indigenous microorganisms, with distinct communities at different anatomical sites^57^. The oral cavity harbored distinctive microbial communities due to its particular ecological and physiological characteristics. Many factors such as the profile of community members, structure of cell wall, physiological state of the cell and relative abundance of cells can affect the representation of specific genomes in the sample preparation and sequencing workflow, which can lead to significant bias in the reconstructed microbiota structure^12,16,58,59^. In this study, in order to characterize the possible bias-generating steps in a complete procedure and to critically assess data quality and comparability of results, we examined potential sources of variation from DNA extraction methods, targeted 16S rRNA hypervariable regions and technical replicates, to quantify the nature and magnitude of their impacts on oral microbiome diversity profiling.

As is recommended by the Microbiome Quality Control Project, a mock community consisting of known typical oral taxa with *a priori* determined ratio was employed so as to provide a golden criterion for validating and comparing the methodological protocols^60^. Adoption of a mock community to assess and calibrate the plethora of experimental and computational methods can be particularly important to microbiome sequencing, as in most circumstances the larger effect size of inter-individual variation than that of methodological variation can hinder identification of those variation specifically attributed to the difference in experimental protocols^28,61^. In this study, the oral taxa selected as constituents of the mock community were typical strains that are implicated in both healthy hosts^62^ and oral inflammations such as dental caries^63^, apical periodontitis^64^ and periodontitis^65^. Moreover, all the strains are Gram-positive bacteria, with cell membranes more resistant to lysis than Gram-negative ones. Therefore, we chose a mock microbiota that consists of difficult-to-extract and abundantly represented oral type strains as a model, to validate the efficiency, robust and reproducibility of the potential bias-generating steps.

A principal concern with current experimental protocols is how to quantitatively assess the sources of variation in microbiota profiling. Our result showed that experimental procedures contributed to up to 98% variation of the microbiota structures, and the variation was mainly originated from DNA extraction method (R^2^ = 0.764), which is in contrast to the hypervariable regions (R^2^ = 0.210). This result was in accordance with those previously reported^39^, which supported the DNA extraction procedure’s critical role in shaping the final DNA yield and sequencing results. However, several studies on human gut microbiome reported the opposite results, i.e., the effect of hypervariable region outweighed that of DNA extraction method on the portrayal of mock community structure^30,37^. The inconsistencies here may arise, *firstly*, from the similarity in the commercial DNA extraction kits that they have compared (in contrast, sample pretreatment prior to DNA extraction was also tested in our study). *Secondly*, in studies that used human oral microbiota rather than the mock communities, the inter-method variation could have been masked by the inter-individual variation^61^.

In our study, all the six extraction methods evaluated offered decent, yet varied, performance on extracting metagenomic DNA from the mock oral microbiota. Higher amounts of DNA yield were obtained from the enzymatic-lysis-only protocol (M3), yet supplementation with [beads beating] or [beads + Lytic- Enzyme Cocktail] yielded a lower amount of DNA. As previous studies reported that the DNA yield was mainly affected by the efficacy of cell lysis, rather than the DNA recovery process^20^, the improvement in DNA extraction efficiency due to use of lysozyme as shown in our study was likely due to the ability of lysozyme to disrupt peptidoglycans in the cell walls from Gram-positive bacteria. Moreover, to some extent, addition of the bead beating step, despite its tendency to lower the DNA yield due to an inevitable loss by inserting an extra processing step, can improve the accuracy of microbiota portrait, as suggested by the sequencing results. Notably, our results are in agreement with other oral^22^ and gut^23^ studies, which observed that the addition of beads plus enzymatic system (M5: lysozyme; M6: a cocktail containing lysozyme, mutanolysin, and lysostaphin) generally produced the most accurate microbial representation. Specifically, a bead-enzyme combination processing step can adequately and accurately identify those Gram-positive bacteria such as *Streptococcus mutans*, thus ensuring a more accurate portrait of the oral microbiota. These results highlighted the importance of appreciating and tracking the potential bias introduced by DNA extraction methods and sample pre-treatment strategies, during the sequencing of oral microbiome samples.

The various 16S hypervariable regions targeted exhibit different degrees of variability. No individual hypervariable regions by itself can discriminate between all known microbial lineages^16^. Therefore, in microbiome sequencing studies, the choice of hypervariable regions may significantly affect the estimates of microbial diversity. Several previous studies have documented considerable variations among targeted 16S rRNA gene regions^17,66^. However, no clear consensus on the hypervariable region choice was reached, as each study has adopted a specific experimental methodology or a variant analysis pipeline, and featured a distinct sample origin, which resulted in the ambiguity in accessing the effect of hypervariable regions on the eventual portrait of oral microbiota. As previously reported^67^ and reaffirmed in this study, the selection of hypervariable regions had a significant impact on the observed oral microbiota structure, while its impact was relatively minor as compared to the DNA extraction methods. Furthermore, although no “perfect” hypervariable region existed from our experimental result^15^, we found that V3-V4 and V4-V5 seemed to produce more reproducible results than V1-V3, which is consistent with the studies on the skin and gut microbiota^30,67^. However, extrapolation of these conclusions to all oral bacteria will require further experiments, as while 16S rRNA gene hypervariable regions exhibited different degrees of variability among species^13,35^, this study only includes five Gram-positive species. Moreover, selection of hypervariable regions produced no obvious variations on the observed microbial diversity, as represented by either Bray-Curtis-distances-based β diversity or the α diversity index values. In our current study, the synthetic oral microbiota of five Gram-positive species is a relatively simple microbiota (relative to, e.g. saliva samples), thus the subtle difference in observed microbial diversity across the three hypervariable regions may in fact be due to the lack of amplification bias among the particular species tested^30^. Nevertheless, the selection of a suitable and relative optimal hypervariable region was of great importance, especially when the goal is to track differences across sampling sites, time scales or treatments, or to compare results obtained by different laboratories.

## Conclusion

The observed oral microbiota structure is highly affected by the choice of DNA extraction method, while the impact of 16S rRNA hypervariable regions is relatively minor. Among the experimental protocols tested, enzymatic-mechanical-lysis based DNA extraction methods performed best on the characterization of oral microbiota diversity. Moreover, V3-V4 and V4-V5 hypervariable regions appeared to lead to more accurate oral-microbiota structure than V1-V3 hypervariable regions. In addition, magnitude of variation in observed microbial diversity was independent of the operators and sequencing batches. In the current study, as the mock community does not fully represent the complex and heterogeneous oral microbiota, our results on the impacts of bias-generated steps including DNA extraction and 16S rRNA hypervariable region on microbiota structure reconstruction should be interpreted within the context of these tested strains, and extrapolation of them to other oral species (particularly Gram-negative organisms) should be treated with caution. Nevertheless, our findings can serve as one reference for scientists in selecting DNA extraction methods and hypervariable regions for their particular research mission.

## Electronic supplementary material

Figure S1
