# Supplementary material for: Impact of DNA extraction method and targeted 16S-rRNA hypervariable region on oral microbiota profiling
Source: Sci Rep. 2018 Nov 5;8:16321. doi: 10.1038/s41598-018-34294-x (PMC6218491; doi:10.1038/s41598-018-34294-x)
Supplement: Supplementary file 1 — Figure S1 [file 41598_2018_34294_MOESM1_ESM.pdf]

Impact of DNA extraction method and targeted 16S-rRNA hypervariable region on oral microbiota profiling

Fei Teng[1, 4,*], Sree Sankar[1, 4], Pengfei Zhu[1], Shanshan Li[2], Shi Huang[1], Xiaolan Li[3], Jian Xu[1] and Fang Yang[2,*]

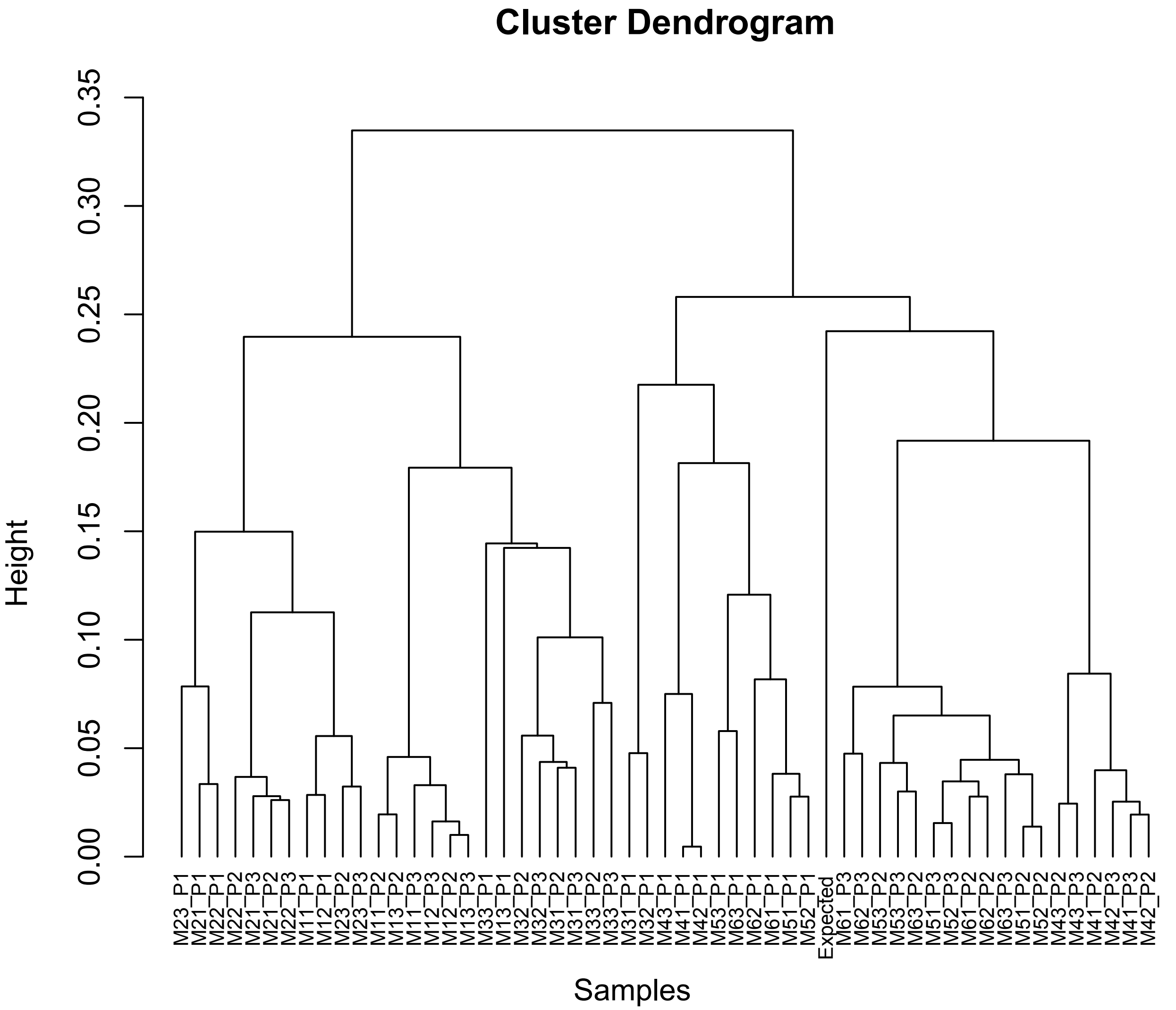
Cluster Dendrogram
Height
0.00
0.05
0.10
0.15
0.20
0.25
0.30
0.35
Samples
M23_P1
M21_P1
M22_P1
M22_P2
M21_P3
M21_P2
M22_P3
M11_P1
M12_P1
M23_P2
M23_P3
M11_P2
M13_P2
M11_P3
M12_P3
M12_P2
M13_P3
M33_P1
M13_P1
M32_P2
M32_P3
M31_P2
M31_P3
M33_P2
M33_P3
M31_P1
M32_P1
M43_P1
M41_P1
M42_P1
M53_P1
M63_P1
M62_P1
M61_P1
M51_P1
M52_P1
Expected
M61_P3
M62_P3
M53_P2
M53_P3
M63_P2
M51_P3
M52_P3
M61_P2
M62_P2
M63_P3
M51_P2
M52_P2
M43_P2
M43_P3
M41_P2
M42_P3
M41_P3
M42_P2

Figure S1. Hierarchical clustering of the microbial profiles obtained using different experimental protocols. Bray–Curtis dissimilarity was calculated using the relative abundance taxa at the species level. Based on the dissimilarity, the hierarchical clustering analysis was performed using the complete linkage algorithm. Protocols that included enzymatic-mechanical-lysis steps in DNA extraction procedure (e.g., M5 and M6) were clustered with the expected microbiome structure, thus they produced better microbial diversity representation than those without the extra steps in the procedure (e.g., M1, M2, and M3). Moreover, samples from P2 (V3_V4) and P3 (V4_V5) tended to cluster closely to the expected microbial structure.
